# Developing a web-based support using self-affirmation to motivate lifestyle changes in type 2 diabetes: A qualitative study assessing patient perspectives on self-management and views on a digital lifestyle intervention

**DOI:** 10.1016/j.invent.2021.100384

**Published:** 2021-03-31

**Authors:** Emelia Mellergård, Per Johnsson, Frida Eek

**Affiliations:** a Department of Health Sciences, Faculty of Medicine, Lund University, Margaretavägen 1B, 222 40 Lund, Sweden; b Department of Psychology, Faculty of Social Sciences, Lund University, Allhelgona kyrkogata 16a, 223 62 Lund, Sweden

**Keywords:** Type 2 diabetes, Self-management, Digital health interventions, Motivation

## Abstract

**Aims:**

The aim of the present study was to explore patients' experiences of diabetes self-management and views on a digital lifestyle intervention using self-affirmation to motivate lifestyle changes.

**Methods:**

Semi-structured interviews focusing on needs, attitudes, and barriers to diabetes self-management were conducted with 22 individuals with type 2 diabetes recruited from the All New Diabetics in Scania (ANDIS) cohort. The interviews were followed by three additional study visits, where participants gave feedback on computer-based assignments based on self-affirmation. Interviews and feedback were qualitatively analyzed using thematic analysis.

**Results:**

Participants described a range of barriers to diabetes self-management, and a varying sense of urgency and distress related to diabetes management. A need for accessible, reliable, and relevant information was reported, as well as a sense that required lifestyle changes was incompatible with current life situation. Further, the use of self-affirmation was described as relevant, motivating and engaging.

**Conclusions:**

Barriers to diabetes self-management need to be addressed when supporting diabetes self-management, e.g. through carefully matching the support to the patient's readiness to change, supporting patient autonomy and focusing on long-term changes. Using self-affirmation may raise acceptability of a digital lifestyle intervention and help connect diabetes self-management with overall life context, by guiding the patient to focus on personal relevance.

## Highlights

• Patients' autonomy needs to be better supported in type 2 diabetes management.
• Self-affirmation can help connect diabetes management with overall life context.
• Both design and content of digital health interventions should be personalized.

## 1 Introduction

Type 2 diabetes is the most common form of diabetes, comprising approximately 95% of all cases of diabetes. It's a condition characterized by chronically elevated blood glucose levels due to an insensitivity to insulin, as well as a low insulin output, which over time can cause a variety of micro- and macrovascular complications (Rubin and Peyrot, 2001). The prevalence of type 2 diabetes is increasing globally, highlighting the need for effective illness management (IDF, 2015).

Standard care for individuals with type 2 diabetes involves advice about lifestyle modification, as a means to keep blood glucose levels close to normal and avoid the risk of diabetic complications. There is evidence supporting that lifestyle changes can prevent or delay the onset of type 2 diabetes, and lifestyle interventions have further been shown to be more effective at reducing the incidence of diabetes than treatment with metformin, the first-line medication for treatment of type 2 diabetes (DPP, 2002; Uusitupa et al., 2019). However, lifestyle changes are difficult to achieve and maintain in the long term (IDF, 2005).

According to self-affirmation theory, people may feel defensive when presented with health messages that require a change of current behavior. When patients are reminded of their unhealthy behaviors, they may reject lifestyle advice as it threatens their sense of self. Supporting individuals to see self-relevance in otherwise threatening health messages, thereby promoting health message receptivity, has been shown to increase physical activity and decrease sedentary behavior (Kang et al., 2018). Hence, in order to have effect on one's lifestyle, health information needs to translate into something of personal value (Pal et al., 2018).

Digital health interventions have the potential to be cost-effective, easy distributable, and personalized to suit patients' needs (Pal et al., 2013; Shan et al., 2019). There are however challenges associated with this type of support, mainly low levels of uptake and reduced user engagement over time (Pal et al., 2018; Kuo et al., 2018). Additionally, acceptance of internet-based interventions tends to be relatively low among patients, raising concerns about their relevance (Bendig et al., 2018). Qualitative research that assesses the patient perspective on these issues can be utilized to develop digital interventions that are both highly relevant and accessible (Pal et al., 2018).

In the present study, patients' attitudes, barriers, and needs towards diabetes self-management were explored, in order to inform the development of a digital lifestyle intervention. A further aim was to address patients' views on design and expectations on content of a digital lifestyle intervention. In order to consider if a focus on self-affirmation could influence acceptability, an additional aim of the study was to describe patients' views on examples of self-affirmation, in the context of diabetes management.

## 2 Materials and methods

### 2.1 Study design

A qualitative research design was employed. Semi-structured interviews covering the experience of having diabetes, barriers to making lifestyle changes, perceived competence of diabetes self-management, previous experience of making lifestyle changes, diabetes self-management support, and goals, were conducted. A total of 22 individuals were interviewed. Following the interviews, participants took part in three additional study visits. During these study visits, the participants were presented with computer-based assignments focusing on self-reflection and viewing current lifestyle in a larger context. The assignments were based on self-affirmation and included identifying personal values, prioritizing among short- and longterm goals, and reflecting on informative texts about diabetes self-management. The assignments were followed by feedback questions to capture participants' thoughts on the use of self-affirmation and on a web-based support in general. Based on the participants' feedback as well as observations of user behavior during the visits, the exercises were iteratively modified by psychologists and physicians in the study team between the visits. The assignments were developed to represent features of a prospective web-based support.

### 2.2 Study setting

The study was conducted at Lund University's Clinical Research Centre in Malmö, Sweden. Patients with type 2 diabetes in the All New Diabetics in Scania (ANDIS) registry who were enrolled in the larger study “Detailed mapping of Type 2 Diabetes” (DIACT) were invited via letters to participate. ANDIS aims to register all incident cases of diabetes within Scania, a Swedish region with more than 1,300,000 inhabitants in both rural and urban areas. Those enrolled in DIACT attended semiannual visits for up to four years for analysis of HbA1c and completion of questionnaires assessing the impact of psychological factors on diabetes self-management, while followed by ordinary healthcare provider.

### 2.3 Study sample

**Fig. 1:**
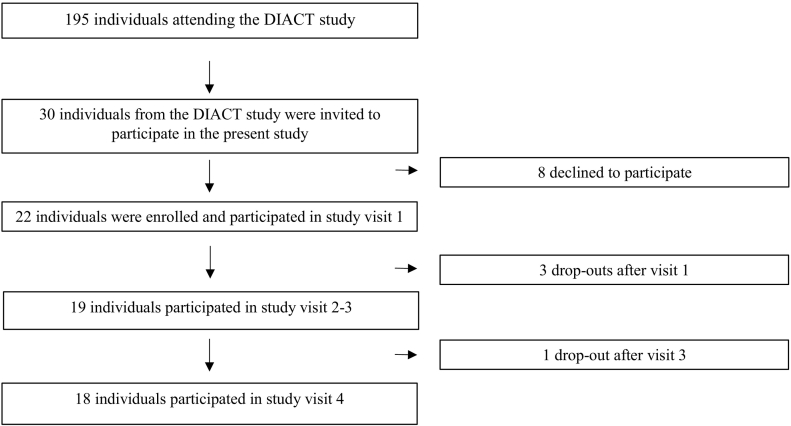
Participant flowchart.

Inclusion criteria were type 2 diabetes diagnosis and age 35–75 years. Exclusion criteria were other endocrine disorders, pregnancy, GAD-antibodies, ongoing medication that could affect blood glucose levels, injury or disease that could affect measurement accuracy or challenge the individual's health upon participation, inability to comprehend the implications of participation in the study, or participation in any other, ongoing study. Participants in the present study were purposefully sampled based on the principles for maximum variation sampling (Patton, 1990). To ensure a diverse sample, a purposively sampling based on age, diabetes duration, treatment, and HbA1c was conducted. A total number of 30 individuals were invited to participate in the interviews. A total of 22 individuals were interested in participation. This number was judged to be appropriate in order to meet the aims of the interviews (Francis et al., 2010). At the time of the interview, further information about the study setup was provided, and the participants' informed consent was collected. Three participants dropped out after the interview and did not participate in the following visits, and one participant dropped out before the last visit because of lack of time (Fig. 1).

### 2.4 Data collection

Data were collected through interviews and feedback on computer-based assignments. All study visits were conducted by the first author, who is a licensed psychologist with extensive knowledge of diabetes and diabetes research. Interviews were conducted during November 2014–January 2015. The interviewer had no prior relationship to the participants. Each interview lasted approximately 1 ½ h. An interview guide was used, covering the experience of having diabetes, barriers to making lifestyle changes, perceived competence of diabetes self-management, previous experience of making lifestyle changes, diabetes self-management support, and goals. The three additional visits following the interview were conducted in February 2015–June 2015. During these visits, participants gave feedback on features of the intended digital lifestyle intervention, as well as their general view on a web-based support for diabetes self-management (Table 1). Interviews were transcribed verbatim, and the feedback from the following visits was summarized in written text. All data were checked against audio recordings for accuracy. To increase credibility, participants were presented with a verbal summary of their interview at the beginning of the second study visit, and were given the opportunity to address any perceived inaccuracies and/or misunderstandings.

**Table 1:** Examples of feedback questions study visit 2–4.

What do you see as the most central parts of a treatment concept as described?
What do you think of the treatment concept in general?
What opportunities or barriers do you see in the treatment concept being computer-based?
Do you believe that this has relevance for your diabetes self-management?
What needs do you see as central to diabetes management and what is needed (by an internet-based treatment concept) to meet them?

### 2.5 Data analysis

Thematic analysis was used to qualitatively analyze the data (Braun and Clarke, 2013). Although thematic analysis can be applied across diverse theoretical approaches, the assumptions underpinning the present study are leaning towards the postpositivist paradigm, i.e. data collection and analysis are attempted to be systematic and transparent, and interpretations derived directly from the data (Denzin and Lincoln, 1994; Guest et al., 2012).

The qualitative data analysis software program ATLAS.ti (version 8.2.4) was used to organize the coding and analysis process. When analyzing the data, overarching themes driven by the research questions were used to sort the data. Data were in this way coded deductively, using the theoretical framework and aim of the study as a starting point to generate initial codes. An inductive approach was then used for coding additional interesting features of the data that could form subthemes. When all data was coded in this way, the different codes were sorted into potential subthemes within the framework. The themes were then reviewed by re-reading the data set, modifying and developing the preliminary themes into main themes by determining what aspects of interest each theme captured. This mode of analyzing can be summarized in five different phases: familiarization, generating initial codes, generating themes, reviewing themes, and defining themes (Braun and Clarke, 2013). The COREQ checklist was consulted when reporting the present study (Tong et al., 2007).

## 3 Results

Of the participants, 12 (54.5%) were men, and 10 (45.5%) were women. Regarding time since diagnosis, 72.7% (*n* = 16), of participants had been diagnosed with diabetes for three years or less, and 63.6% (*n* = 14) were prescribed metformin. One participant was prescribed insulin. Mean age was 68.7 years (SD 8.1, range 44–78), and 18 (81.8%) individuals were married or cohabiting. Of the participants, 7 (31.8%) individuals had completed elementary school as highest education, 5 (22.7%) had completed secondary school, and 10 (45.5%) had a university education.

### 3.1 Attitudes towards making lifestyle changes

Participants described a varying sense of acuteness regarding making lifestyle changes that would benefit their health. Some reported feeling worried that the diabetes would get worse, that diabetes was regarded as a “death sentence”, and that getting diabetes was unexpected, others reported not feeling troubled or restricted by having diabetes. Participants with other health conditions described diabetes as the lesser of their illnesses, negatively affecting their motivation to engage in diabetes management. Not considering diabetes as particularly acute or something to worry about was expressed in the following ways:

“*I am aware that I have it and I try to take certain measures, like I said, with diet and exercise, but otherwise it's nothing to worry about.*”

[Participant 2]

*As an illness, it is one of my minor concerns, it doesn't manifest itself in a severe way. I become, well, I've been tired for many years now, but I'm probably getting a little more tired because of it. But that's probably the only way I notice it.*

[Participant 3]

Not knowing what to do if one's diabetes got worse was a recurring topic, and beliefs about good self-management was expressed in terms of stability and control. Participants expressed positive beliefs about their own ability to manage diabetes, although some were unsure if their efforts were enough or what information about diabetes management to trust.

*It… If it is as it is now, it doesn't feel like a problem. But if something changes or gets worse, then I have no idea, because I haven't been through that. I can't know.*

[Participant 3]

*I don't worry about this type 2 diabetes, because I tell myself that I can manage it so it stays where it is now (…) So I don't worry for a second. I have mentally tuned in to, I think, that I can influence it myself.*

[Participant 6]

Participants also described difficulties identifying themselves with making lifestyle changes that would benefit their health, and experienced a disconnect between their way of life and what they felt were required from them in order to manage diabetes properly:

*I'm not a person who enjoys sports. And they say you have to go out running. No, I don't. My body has been sitting still on a chair since I became an adult, it would die if I went out in the running track. So I don't.*

[Participant 4]

### 3.2 Barriers towards making lifestyle changes

Lack of motivation was reported as a common barrier when attempting to make lifestyle changes. Participants described a variety of reasons for lacking motivation, including not noticing any results (e.g. improved glycemic control, lower weight), insufficient personalization of lifestyle advice, the prioritization of other things, and the feeling that lifestyle changes were not urgent enough. Some participants reported that they were aware of the changes they needed to do, but that it would be too much of a sacrifice to implement these changes long-term.

*I don't have enough motivation for my diabetes to avoid these things all year long, because I think that life is not worth living then. If you can't treat yourself a little extra.*

[Participant 2]

*It's like smoking, what's stopping you from quitting? Well, that you want to continue. You want to eat good (…). I know it's not good for me. So it shouldn't be that hard to do less of it. You don't want to quit completely.*

[Participant 11]

Both physical and emotional barriers were reported as barriers towards optimal diabetes self-management. Among physical barriers were other health issues, pain, older age, or lack of physical energy. Emotional barriers included feeling stressed, not feeling listened to by health care professionals, and feeling that the proposed lifestyle changes were incompatible with one's current lifestyle. These barriers were described as causing additional distress about having type 2 diabetes.

*Mentally too. That hopelessness or meaninglessness sort of take over. It's like, “What the hell am I going to do” (…)*

[Participant 3]

Participants also described a struggle to overcome previous lifestyle habits, in favor of healthier habits. Among the habits described were sedentariness, irregular meals, and a short-term focus that may prevent the individual from establishing sustainable, long-term routines.

### 3.3 Needs concerning making lifestyle changes

Participants expressed the need for more information regarding the daily management of diabetes, and difficulties knowing what information to trust. A general lack of knowledge regarding type 2 diabetes was reported, including not having enough information about issues concerning diabetes risks, glycemic control, insulin, and blood glucose values. Participants further expressed a need for both professional support and support from significant others. The professional support was described in terms of information, feedback, and encouragement in order to support autonomy, and the support from significant others was primarily described as emotional.

### 3.4 Participants' views on a web-based tool targeting lifestyle changes: utility, design, and expectations on content

The themes that were generated relating to the utility of a web-based tool were *Encouragement*, *Information*, and *Prioritization* (Table 2). Participants described that they would like to use a web-based tool for encouragement, both social and professional. Getting information was further reported as a way participants imagined they would utilize a web-based support. This included getting practical help and advice regarding type 2 diabetes and self-management of type 2 diabetes. Getting information was described as motivating, as a way to decrease distress, and as a practical help in making lifestyle changes that would benefit health. Some participants expressed a wish for easy access to professional support. Participants also described that they would like to use a web-based tool as a help to prioritize, e.g. by setting goals aligned with one's own values and needs, and as a support to notice associations between one's own habits and glycemic control.

**Table 2:** Participants' descriptions of how they would use a web-based tool to support lifestyle changes.

Encouragement	Information	Prioritization
Contact with others in the same situation	Getting answers to diabetes related questions	Help setting goals
Contact with health care professionals	Access to reliable information about type 2 diabetes and diabetes self-management	Gaining understanding of the seriousness of type 2 diabetes in order to make healthy choices
Sharing experiences of diabetes with others	Tracking of one's own blood glucose values and blood pressure	Being reminded about one's important values
Receiving encouragement from others		Help to understand what one needs to change when it comes to lifestyle habits
		Support to notice associations between one's own habits and blood glucose values

Participants' views on design of the web-based tool were categorized into two themes: *Accessible* and *Reliable* (Table 3). Participants described a variety of ways in which they would like the web-based tool to be accessible to them. Making the tool easy to navigate was a recurring topic, with different suggestions on how this could be accomplished: not having too many features, possible to quickly get an overview, easy to understand what's what, and having short explanations of different features. Participants additionally had suggestions on making the tool more accessible by personalizing it. This included using one's own name and being addressed in a personal way when using the tool. Participants further emphasized the importance of the web-based tool to feel reliable, including providing reliable information. Continuously updating information was also reported as important, to ensure the relevance of the tool.

**Table 3:** Participants' suggestions on design of a web-based tool to support lifestyle changes.

Accessible	Reliable
Easy to navigate: not too many features, not too complicated, easy to get an overview, adaptable for those with poor eyesight, easy to know what's what, short explanations/tutorials for the different features,	Up-to-date information
Able to use on mobile phone or tablet, not only computer	Information about who's behind the tool
Personalized	Regular updates
Possible for users to give feedback on things that are not working	A layout that gives a “serious” impression

Themes associated with participants' descriptions of their expectations on content were *Track changes*, *Set goals*, and *Personalization* (Table 4). The possibility to track one's progress or notice deviations was expressed as an important feature. Some described the usefulness of different graphical presentations and comparisons, in order to explore associations between lifestyle related factors. Participants further described that they would like to be able to use the web-based support as a tool to plan and motivate them to carry through lifestyle changes. Some expected a feature to evaluate goal attainment, in order to determine what has worked and not. Participants also emphasized that the focus should be on possibilities, rather than on giving pointers and telling you what to do. Additionally, a personalization of the web-based tool was described as an important feature, e.g. by providing tailored information to each user. Tailored information was described as practical tips and advice adapted to one's life situation, rather than general information. Some also expressed a wish for help with stress management.

**Table 4:** Examples of participants' expectations on content on a web-based tool to support lifestyle changes.

Track changes	Set goals	Personalization
Graphical presentations	Tool for planning, e.g. calendar	Tailored information
Tool to register blood glucose values, weight and body measures, dietary intake, and physical activity	The possibility to evaluate goals and goal attainment	Practical tips and advice
The possibility to see if there is any association between for example diet and blood glucose values	A focus on possibilities rather than someone telling you what to do	Help with individual needs related to diabetes management, e.g. stress management
		Possibility to get in touch with health care professionals

### 3.5 Participants' views on examples of self-affirmation in the context of diabetes management

Participants described ways in which the assignments helped them gain personal insight through reflection, for example by making them step out of their comfort zone, providing help to search within themselves and be self-critical (as opposed to solely focusing on their ideal self), and to think outside the box. Some found it difficult and too personal, while others reported that it prompted thoughts about what's important in life, which was described as a positive experience. One participant described that the assignments reminds you that you should do the most of life and focus on your own responsibility, and another participant described that the assignments can help you reflect upon your diabetes and normalize it to decrease feelings of distress. Participants further highlighted the value of being reminded about their priorities and having a long-term perspective in the management of diabetes. Reflecting on what's important and what to focus on, was described as a help to keep continuity and to connect ideas about making lifestyle changes to everyday life. Assignments were evaluated as both relevant and useful to diabetes management, eliciting thoughts about one's ability to make choices and the decisions you could make in order to live a healthy life. Supporting participants to put their own words on things they would like to be different were described as helpful. Acting as a reminder of one's attitudes, values, and life choices, assignments were additionally reported being a source of motivation and inspiration to the participants. Some participants were unsure of how the assignments connected to diabetes management, although this was not reported as a reason not to engage with the assignments, which were described as inspiring and valuable.

## 4 Discussion

The results acknowledge that the experience of having diabetes is highly heterogeneous, and that diabetes distress may influence the readiness to make lifestyle changes. Diabetes distress, the negative emotional experience resulting from the challenge of living with the demands of diabetes, is associated with poorer self-management (Fisher et al., 2019). Previous studies have shown that diabetes distress, as well as perceived competence to manage diabetes, is associated with glycemic control in individuals with type 2 diabetes (Mellergård et al., 2020). Results from the present study further emphasize the need for individualized feedback and emotional support. Although lifestyle interventions have been found to be effective in clinical trials, their efficacy in a real world setting is primarily dependent upon the engagement of the individual (Fisher et al., 2017). Solely focusing on standard information provision and the medical management of diabetes may not meet all the needs of patients with type 2 diabetes, and there is no good evidence of a link between knowledge and adherence in diabetes management (Pal et al., 2018; Harvey and Lawson, 2009). This perspective also needs to be incorporated into digital health interventions addressing diabetes management. In the present study, participants expressed a lack of identification with making lifestyle changes that would benefit their health, stressing the importance of the patient's role in designing changes that fit one's life situation. Patient autonomy is associated with enhanced motivation, internalization and long-term change of behavior, as well as better glycemic control (Williams et al., 2004; Deci and Ryan, 2008; Teixeira et al., 2012). In spite of this, support for patient independence has been shown to be lacking in diabetes care and instead control is favored: checking the patients' laboratory tests and recommending treatment strategies in order to achieve appropriate results (Schimmer et al., 2019). In order to promote motivation and personal engagement, the results from the present study suggest that the patient needs to be supported to take the leading part of diabetes management.

Additionally, results indicate that patients may find it difficult to find accessible, reliable and relevant information about diabetes management. Participants in the present study further had several expectations on a web-based support, such as the possibility to track changes, set goals, and have access to tailored information. These results are in line with previous recommendations that web-based supports should adapt both design and content to match the users' preferences (Schimmer et al., 2019; Kelly et al., 2018).

Previous studies have shown that more emphasis should be placed on enabling retention when developing web-based interventions, since attrition rates are frequently high. Even the most effective program will not have impact on health outcomes if its actual usage is low (Kelly et al., 2018; Leung et al., 2017). Participants in the present study reported the use of self-affirmation as an overall positive and motivating experience, suggesting that an emphasis on personal values, meaning, priorities, and “the bigger picture”, possibly could enhance acceptability through its focus on personal relevance.

### 4.1 Strengths and limitations

The extent of the study, encompassing four separate visits with each participant, and the utilization of participant checks in order to validate the interview data, contributes to the study's credibility, i.e. the rigor of the research process. In qualitative approaches, credibility can be referred to as internal validity (Morrow, 2005). The external validity, or the study's transferability, was in the present study assessed by providing detailed information about the study context, process, participants, and researcher-participant relationship.

The analysis in the present study was conducted by one of the authors, although regularly reviewed and discussed with the other researchers involved. Having several analysts could strengthen the claims made, since researcher triangulation could have enabled a broader and more complex understanding of the phenomena (Tong et al., 2007). However, a detailed chronology of research activities and processes, such as emerging themes, was kept during the work with the present study, ensuring that the process through which the findings were derived may to some extent be repeatable (Morrow, 2005). Participants in the present study were purposefully sampled based on the principles for maximum variation sampling, to include a diverse sample based on age, diabetes duration, HbA1c, and lifestyle changes made when diagnosed with diabetes. Maximum variation sampling is suitable when sampling smaller samples from heterogenous groups, in order to ensure that the variation from the larger group is kept in the smaller sample, i.e. to warrant that information-rich cases are included in the study sample (Patton, 1990). This methodological approach was considered appropriate in order to include a diverse sample that could represent the highly heterogenous group that individuals with type 2 diabetes are.

## 5 Conclusions

Results show that different barriers to diabetes management need to be addressed when supporting diabetes self-management, e.g. through carefully matching the support to the patient's readiness to change, and enhancing patient autonomy. When developing a web-based support, patients would like to have the possibility to track changes, set goals, and have access to tailored information, and both design and content should signal seriousness, relevance, and personalization. Additionally, using self-affirmation could raise motivation and acceptability by guiding the patient to focus on personal relevance. Future studies may use the insights from this study when developing and evaluating digital health interventions supporting diabetes management, in order to make them relevant and increase acceptability.

## Declaration of competing interest

The authors declare that they have no known competing financial interests or personal relationships that could have appeared to influence the work reported in this paper.
